# Supplementary material for: MetaRibo-Seq measures translation in microbiomes
Source: Nat Commun. 2020 Jun 29;11:3268. doi: 10.1038/s41467-020-17081-z (PMC7324362; doi:10.1038/s41467-020-17081-z)
Supplement: Supplementary file 10 — Supplementary Data 7 [file 41467_2020_17081_MOESM10_ESM.zip › File2/Confidence_High_Taxonomy/88895_out.krona.html]

Javascript must be enabled to view this page.

members
magnitude
magnitudeUnassigned
count
unassigned
taxon
rank

88895\_out

5

superkingdom
2
5

1224
5
phylum

5
28216
class

order
206351
5

481
5
family

genus
5
32257


SRS017445\_contig\_number\_17374SRS020406\_contig\_number\_3292SRS053584\_contig\_number\_6979SRS147128\_contig\_number\_contig-100\_746.78811SRS149647\_contig\_number\_3097
5
502
species
